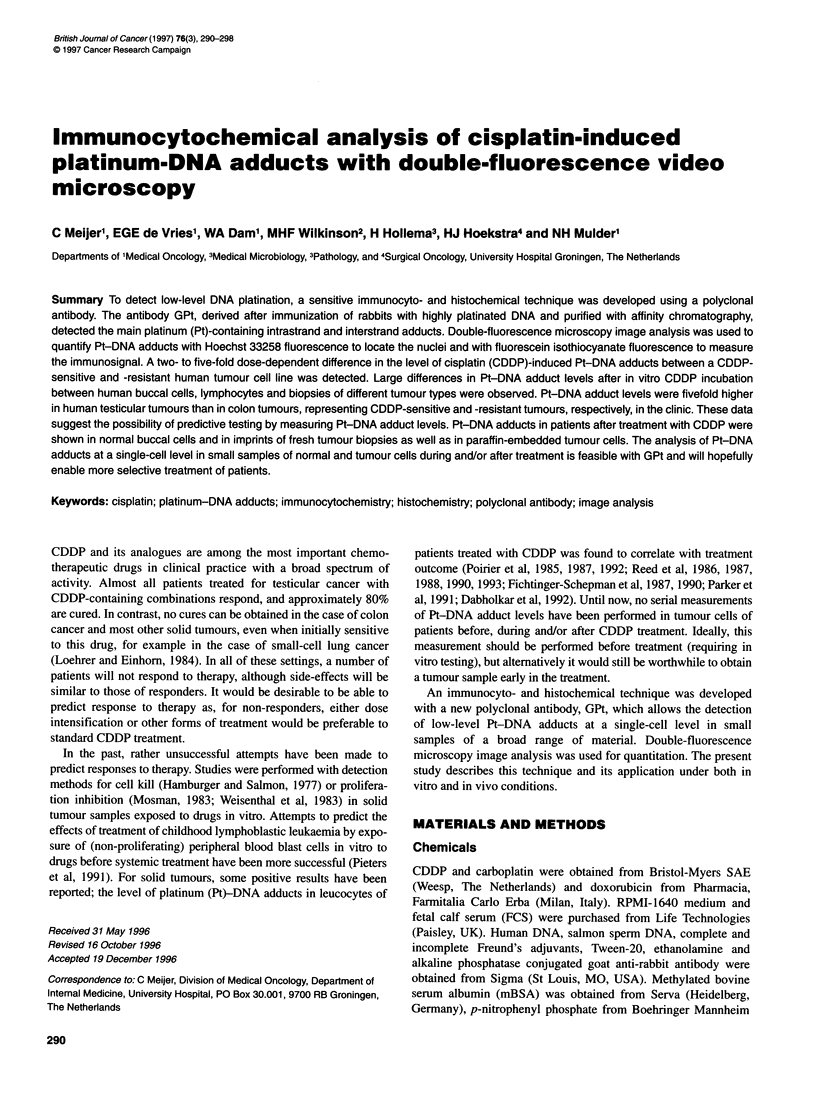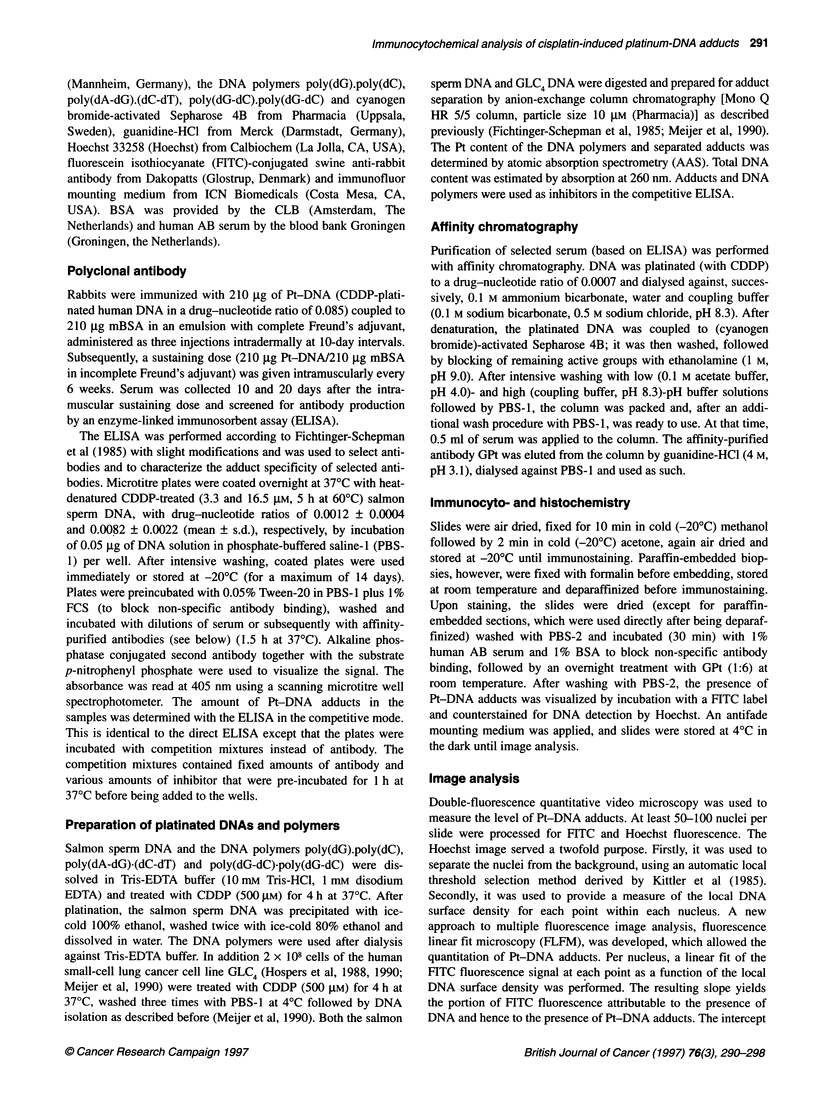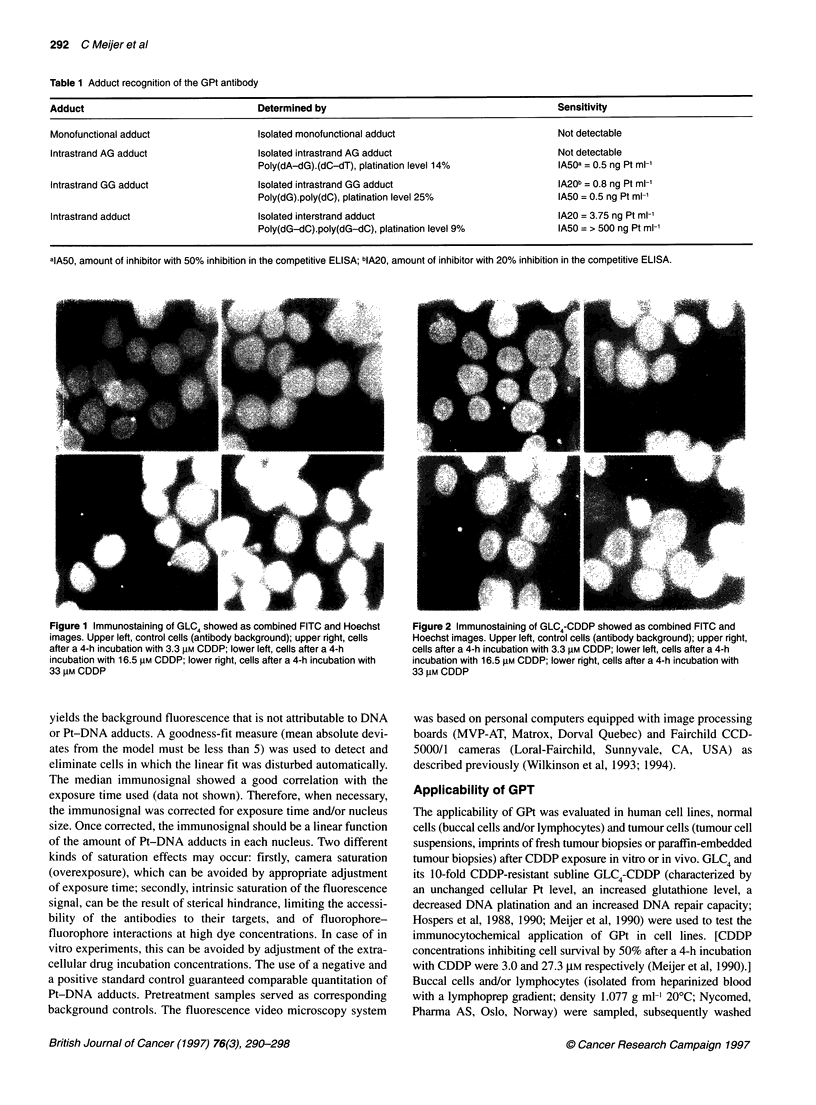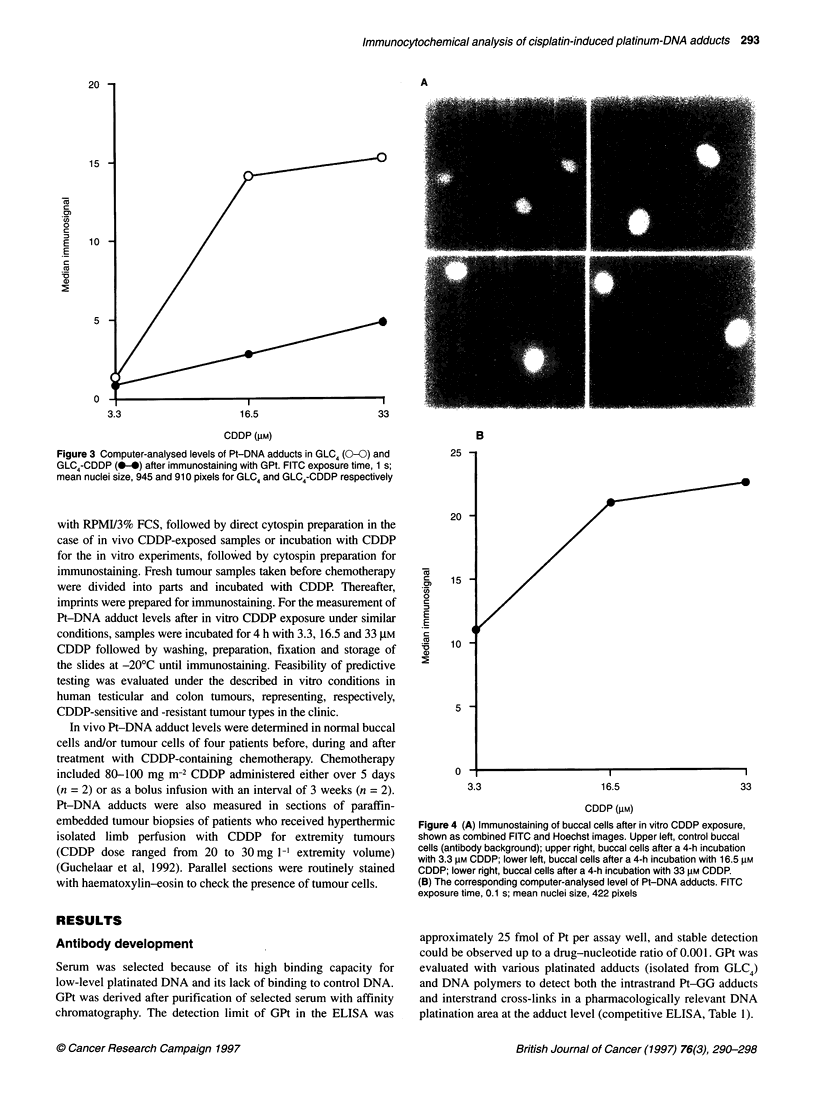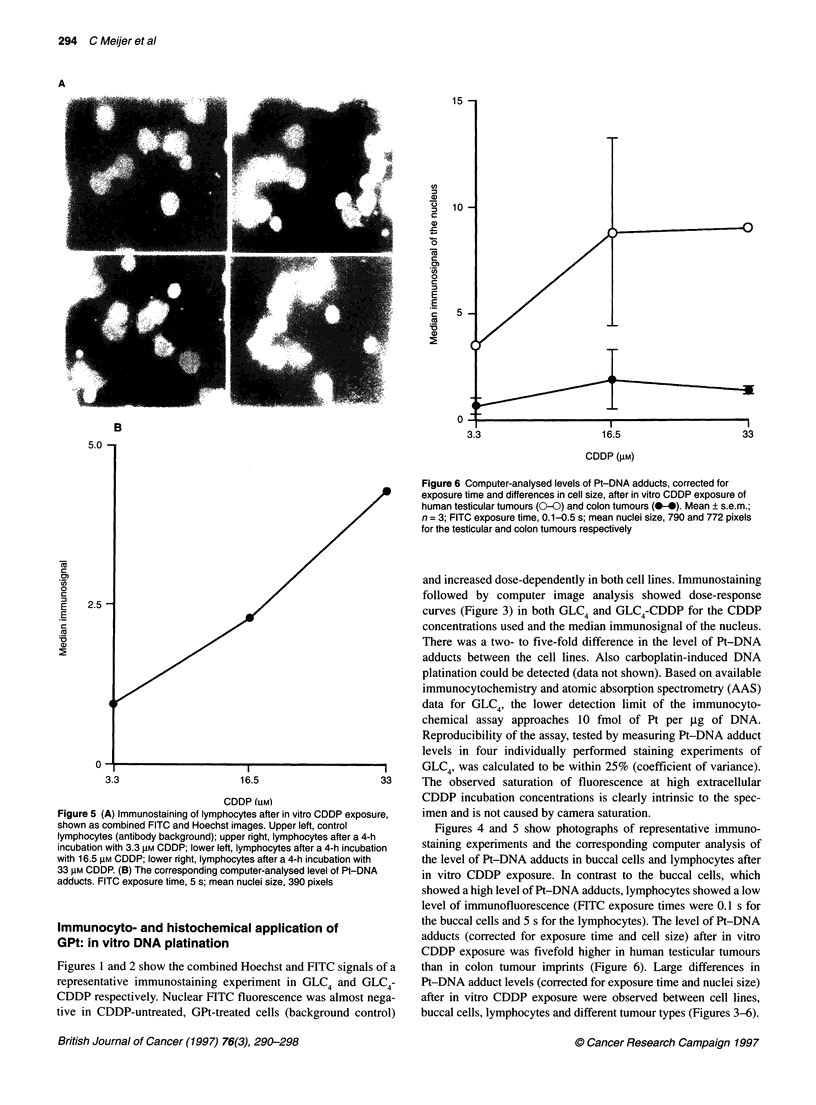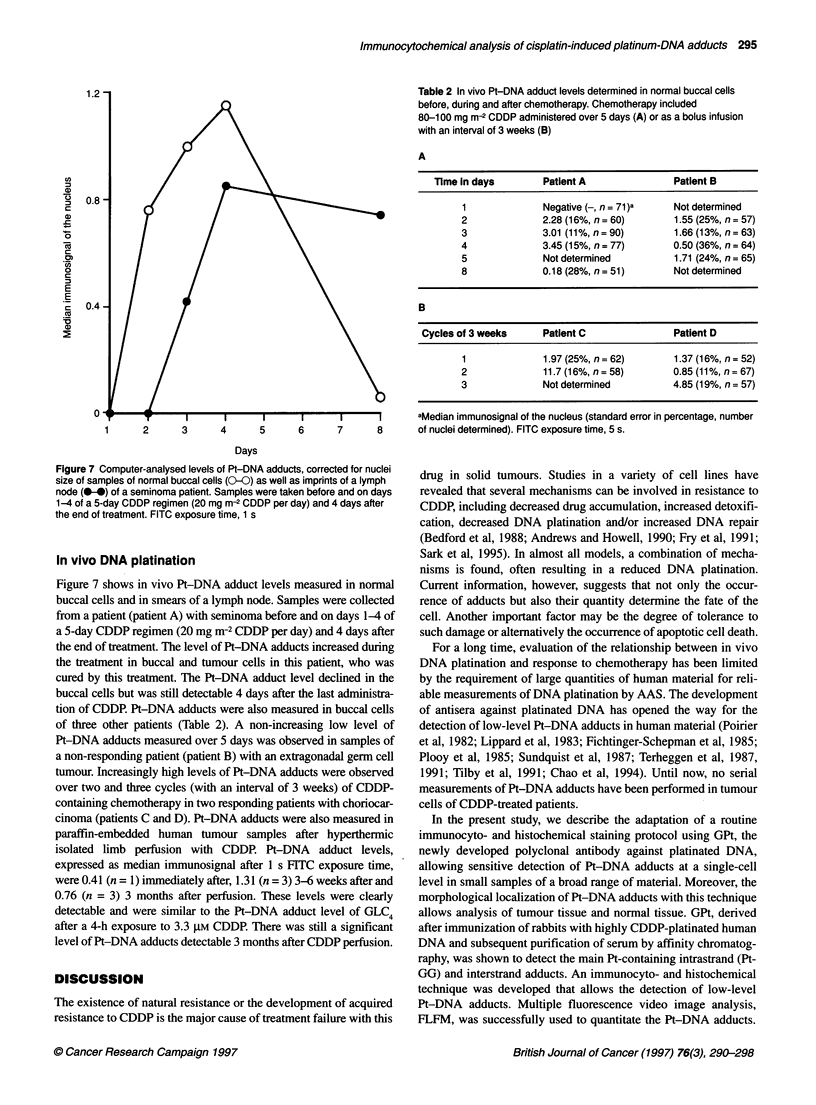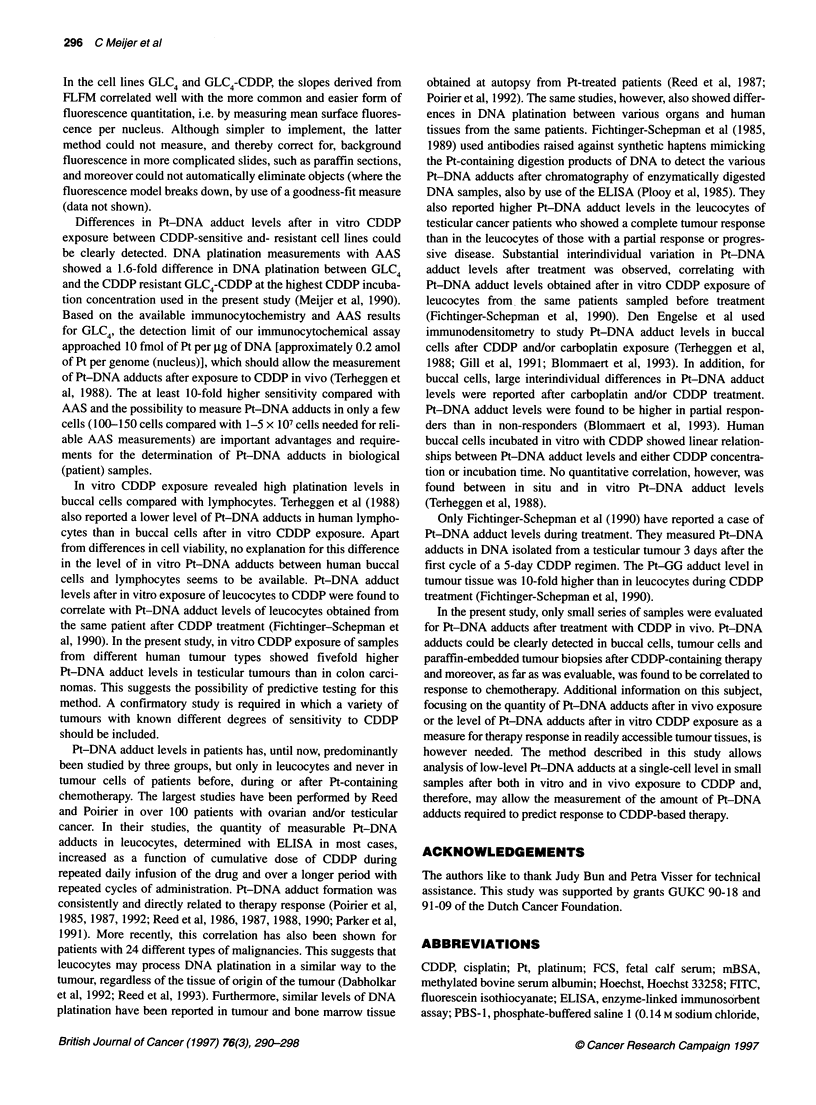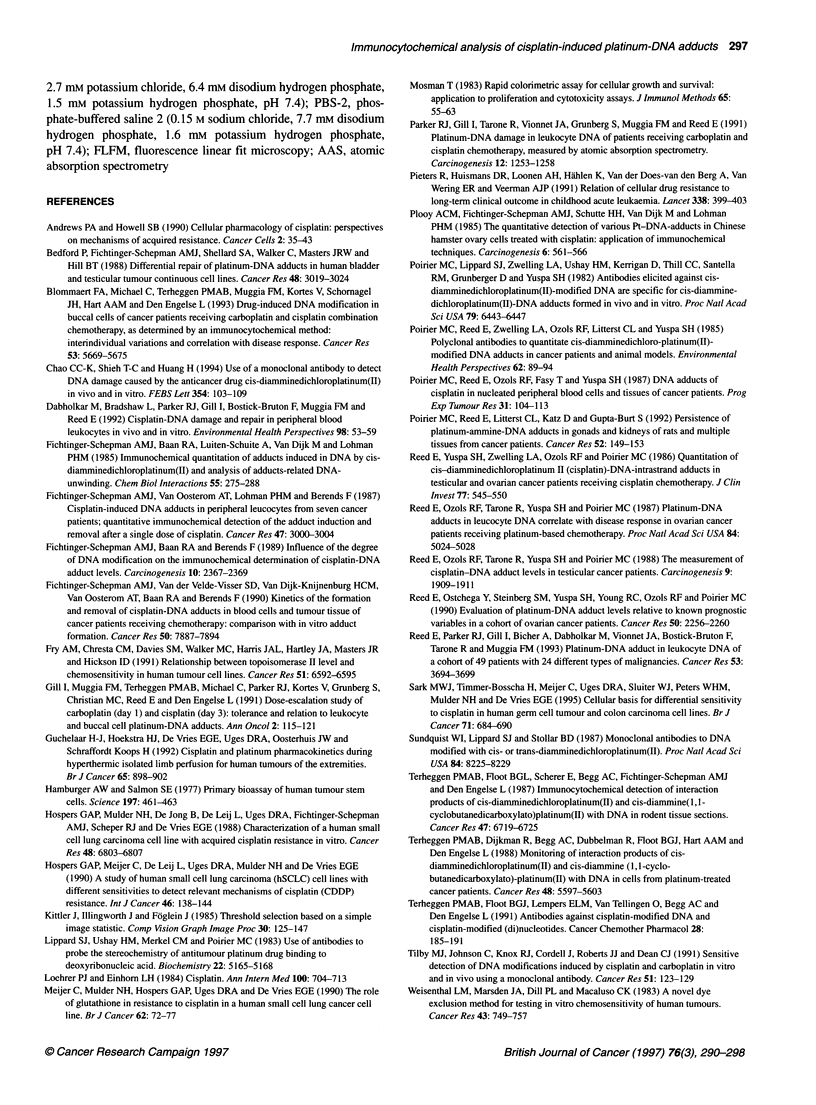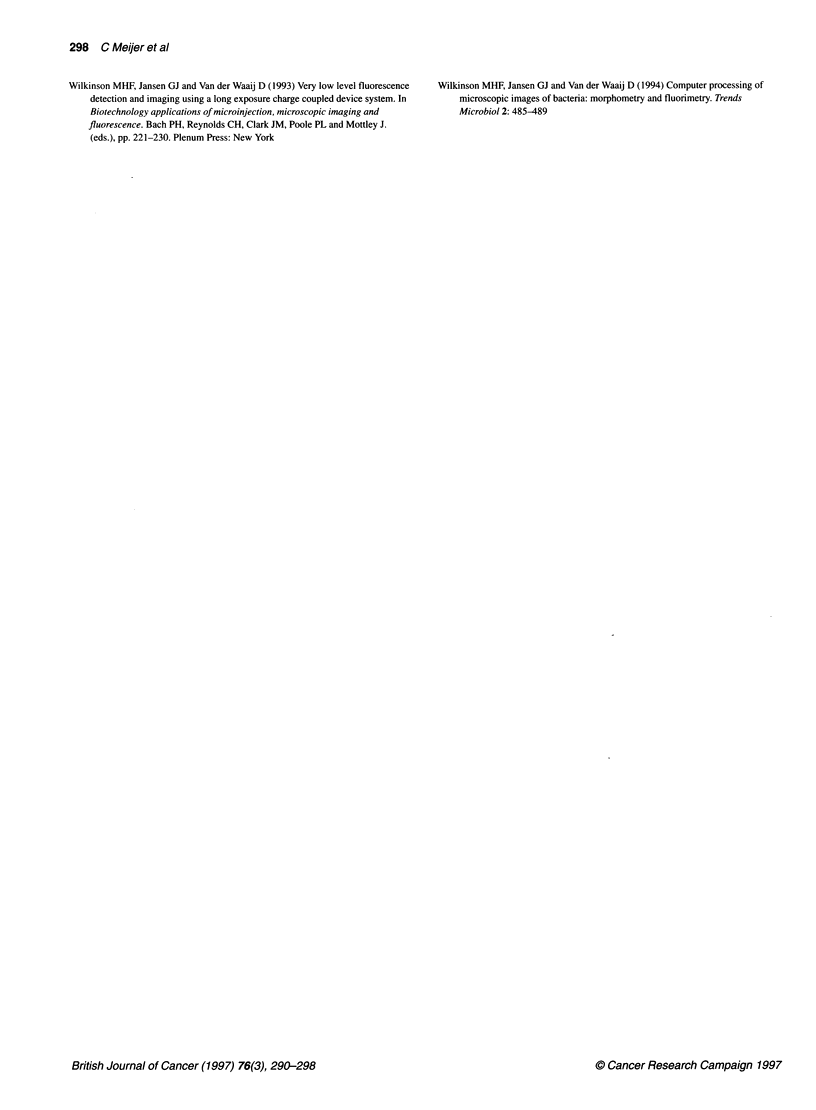# Immunocytochemical analysis of cisplatin-induced platinum-DNA adducts with double-fluorescence video microscopy.

**DOI:** 10.1038/bjc.1997.381

**Published:** 1997

**Authors:** C. Meijer, E. G. de Vries, W. A. Dam, M. H. Wilkinson, H. Hollema, H. J. Hoekstra, N. H. Mulder

**Affiliations:** Department of Medical Oncology, University Hospital Groningen, The Netherlands.

## Abstract

**Images:**


British Journal of Cancer (1997) 76(3), 290–298
© 1997 Cancer Research Campaign

### Immunocytochemical analysis of cisplatin-induced platinum-DNA adducts with double-fluorescence video microscopy

**C Meijer[1], EGE de Vries[1], WA Dam[1], MHF Wilkinson[2], H Hollema[3], HJ Hoekstra[4] and NH Mulder[1]**

Departments of [1]Medical Oncology, [3]Medical Microbiology, [3]Pathology, and [4]Surgical Oncology, University Hospital Groningen, The Netherlands

**Summary** To detect low-level DNA platination, a sensitive immunocyto- and histochemical technique was developed using a polyclonal antibody. The antibody GPt, derived after immunization of rabbits with highly platinated DNA and purified with affinity chromatography, detected the main platinum (Pt)-containing intrastrand and interstrand adducts. Double-fluorescence microscopy image analysis was used to quantify Pt–DNA adducts with Hoechst 33258 fluorescence to locate the nuclei and with fluorescein isothiocyanate fluorescence to measure the immunosignal. A two- to five-fold dose-dependent difference in the level of cisplatin (CDDP)-induced Pt–DNA adducts between a CDDP-sensitive and -resistant human tumour cell line was detected. Large differences in Pt–DNA adduct levels after in vitro CDDP incubation between human buccal cells, lymphocytes and biopsies of different tumour types were observed. Pt–DNA adduct levels were fivefold higher in human testicular tumours than in colon tumours, representing CDDP-sensitive and -resistant tumours, respectively, in the clinic. These data suggest the possibility of predictive testing by measuring Pt–DNA adduct levels. Pt–DNA adducts in patients after treatment with CDDP were shown in normal buccal cells and in imprints of fresh tumour biopsies as well as in paraffin-embedded tumour cells. The analysis of Pt–DNA adducts at a single-cell level in small samples of normal and tumour cells during and/or after treatment is feasible with GPt and will hopefully enable more selective treatment of patients.

**Keywords:** cisplatin; platinum–DNA adducts; immunocytochemistry; histochemistry; polyclonal antibody; image analysis

CDDP and its analogues are among the most important chemotherapeutic drugs in clinical practice with a broad spectrum of activity. Almost all patients treated for testicular cancer with CDDP-containing combinations respond, and approximately 80% are cured. In contrast, no cures can be obtained in the case of colon cancer and most other solid tumours, even when initially sensitive to this drug, for example in the case of small-cell lung cancer (Loehrer and Einhorn, 1984). In all of these settings, a number of patients will not respond to therapy, although side-effects will be similar to those of responders. It would be desirable to be able to predict response to therapy as, for non-responders, either dose intensification or other forms of treatment would be preferable to standard CDDP treatment.

In the past, rather unsuccessful attempts have been made to predict responses to therapy. Studies were performed with detection methods for cell kill (Hamburger and Salmon, 1977) or proliferation inhibition (Mosman, 1983; Weisenthal et al, 1983) in solid tumour samples exposed to drugs in vitro. Attempts to predict the effects of treatment of childhood lymphoblastic leukaemia by exposure of (non-proliferating) peripheral blood blast cells in vitro to drugs before systemic treatment have been more successful (Pieters et al, 1991). For solid tumours, some positive results have been reported; the level of platinum (Pt)–DNA adducts in leucocytes of patients treated with CDDP was found to correlate with treatment outcome (Poirier et al, 1985, 1987, 1992; Reed et al, 1986, 1987, 1988, 1990, 1993; Fichtinger-Schepman et al, 1987, 1990; Parker et al, 1991; Dabholkar et al, 1992). Until now, no serial measurements of Pt–DNA adduct levels have been performed in tumour cells of patients before, during and/or after CDDP treatment. Ideally, this measurement should be performed before treatment (requiring in vitro testing), but alternatively it would still be worthwhile to obtain a tumour sample early in the treatment.

An immunocyto- and histochemical technique was developed with a new polyclonal antibody, GPt, which allows the detection of low-level Pt–DNA adducts at a single-cell level in small samples of a broad range of material. Double-fluorescence microscopy image analysis was used for quantitation. The present study describes this technique and its application under both in vitro and in vivo conditions.

*Received 31 May 1996*
*Revised 16 October 1996*
*Accepted 19 December 1996*

*Correspondence to:* C Meijer, Division of Medical Oncology, Department of Internal Medicine, University Hospital, PO Box 30.001, 9700 RB Groningen, The Netherlands

### MATERIALS AND METHODS

### Chemicals

CDDP and carboplatin were obtained from Bristol-Myers SAE (Weesp, The Netherlands) and doxorubicin from Pharmacia, Farmitalia Carlo Erba (Milan, Italy). RPMI-1640 medium and fetal calf serum (FCS) were purchased from Life Technologies (Paisley, UK). Human DNA, salmon sperm DNA, complete and incomplete Freund's adjuvants, Tween-20, ethanolamine and alkaline phosphatase conjugated goat anti-rabbit antibody were obtained from Sigma (St Louis, MO, USA). Methylated bovine serum albumin (mBSA) was obtained from Serva (Heidelberg, Germany), *p*-nitrophenyl phosphate from Boehringer Mannheim

(Mannheim, Germany), the DNA polymers poly(dG).poly(dC), poly(dA-dG).(dC-dT), poly(dG-dC).poly(dG-dC) and cyanogen bromide-activated Sepharose 4B from Pharmacia (Uppsala, Sweden), guanidine-HCl from Merck (Darmstadt, Germany), Hoechst 33258 (Hoechst) from Calbiochem (La Jolla, CA, USA), fluorescein isothiocyanate (FITC)-conjugated swine anti-rabbit antibody from Dakopatts (Glostrup, Denmark) and immunofluor mounting medium from ICN Biomedicals (Costa Mesa, CA, USA). BSA was provided by the CLB (Amsterdam, The Netherlands) and human AB serum by the blood bank Groningen (Groningen, the Netherlands).

### Polyclonal antibody

Rabbits were immunized with 210 µg of Pt–DNA (CDDP-platinated human DNA in a drug–nucleotide ratio of 0.085) coupled to 210 µg mBSA in an emulsion with complete Freund's adjuvant, administered as three injections intradermally at 10-day intervals. Subsequently, a sustaining dose (210 µg Pt–DNA/210 µg mBSA in incomplete Freund's adjuvant) was given intramuscularly every 6 weeks. Serum was collected 10 and 20 days after the intramuscular sustaining dose and screened for antibody production by an enzyme-linked immunosorbent assay (ELISA).

The ELISA was performed according to Fichtinger-Schepman et al (1985) with slight modifications and was used to select antibodies and to characterize the adduct specificity of selected antibodies. Microtitre plates were coated overnight at 37°C with heat-denatured CDDP-treated (3.3 and 16.5 µM, 5 h at 60°C) salmon sperm DNA, with drug–nucleotide ratios of 0.0012 ± 0.0004 and 0.0082 ± 0.0022 (mean ± s.d.), respectively, by incubation of 0.05 µg of DNA solution in phosphate-buffered saline-1 (PBS-1) per well. After intensive washing, coated plates were used immediately or stored at –20°C (for a maximum of 14 days). Plates were preincubated with 0.05% Tween-20 in PBS-1 plus 1% FCS (to block non-specific antibody binding), washed and incubated with dilutions of serum or subsequently with affinity-purified antibodies (see below) (1.5 h at 37°C). Alkaline phosphatase conjugated second antibody together with the substrate *p*-nitrophenyl phosphate were used to visualize the signal. The absorbance was read at 405 nm using a scanning microtitre well spectrophotometer. The amount of Pt–DNA adducts in the samples was determined with the ELISA in the competitive mode. This is identical to the direct ELISA except that the plates were incubated with competition mixtures instead of antibody. The competition mixtures contained fixed amounts of antibody and various amounts of inhibitor that were pre-incubated for 1 h at 37°C before being added to the wells.

### Preparation of platinated DNAs and polymers

Salmon sperm DNA and the DNA polymers poly(dG).poly(dC), poly(dA-dG)·(dC-dT) and poly(dG-dC)·poly(dG-dC) were dissolved in Tris-EDTA buffer (10 mM Tris-HCl, 1 mM disodium EDTA) and treated with CDDP (500 µM) for 4 h at 37°C. After platination, the salmon sperm DNA was precipitated with ice-cold 100% ethanol, washed twice with ice-cold 80% ethanol and dissolved in water. The DNA polymers were used after dialysis against Tris-EDTA buffer. In addition $2 \times 10^8$ cells of the human small-cell lung cancer cell line $GLC_4$ (Hospers et al, 1988, 1990; Meijer et al, 1990) were treated with CDDP (500 µM) for 4 h at 37°C, washed three times with PBS-1 at 4°C followed by DNA isolation as described before (Meijer et al, 1990). Both the salmon sperm DNA and $GLC_4$ DNA were digested and prepared for adduct separation by anion-exchange column chromatography [Mono Q HR 5/5 column, particle size 10 µM (Pharmacia)] as described previously (Fichtinger-Schepman et al, 1985; Meijer et al, 1990). The Pt content of the DNA polymers and separated adducts was determined by atomic absorption spectrometry (AAS). Total DNA content was estimated by absorption at 260 nm. Adducts and DNA polymers were used as inhibitors in the competitive ELISA.

### Affinity chromatography

Purification of selected serum (based on ELISA) was performed with affinity chromatography. DNA was platinated (with CDDP) to a drug–nucleotide ratio of 0.0007 and dialysed against, successively, 0.1 M ammonium bicarbonate, water and coupling buffer (0.1 M sodium bicarbonate, 0.5 M sodium chloride, pH 8.3). After denaturation, the platinated DNA was coupled to (cyanogen bromide)-activated Sepharose 4B; it was then washed, followed by blocking of remaining active groups with ethanolamine (1 M, pH 9.0). After intensive washing with low (0.1 M acetate buffer, pH 4.0)- and high (coupling buffer, pH 8.3)-pH buffer solutions followed by PBS-1, the column was packed and, after an additional wash procedure with PBS-1, was ready to use. At that time, 0.5 ml of serum was applied to the column. The affinity-purified antibody GPt was eluted from the column by guanidine-HCl (4 M, pH 3.1), dialysed against PBS-1 and used as such.

### Immunocyto- and histochemistry

Slides were air dried, fixed for 10 min in cold (–20°C) methanol followed by 2 min in cold (–20°C) acetone, again air dried and stored at –20°C until immunostaining. Paraffin-embedded biopsies, however, were fixed with formalin before embedding, stored at room temperature and deparaffinized before immunostaining. Upon staining, the slides were dried (except for paraffin-embedded sections, which were used directly after being deparaffinized) washed with PBS-2 and incubated (30 min) with 1% human AB serum and 1% BSA to block non-specific antibody binding, followed by an overnight treatment with GPt (1:6) at room temperature. After washing with PBS-2, the presence of Pt–DNA adducts was visualized by incubation with a FITC label and counterstained for DNA detection by Hoechst. An antifade mounting medium was applied, and slides were stored at 4°C in the dark until image analysis.

### Image analysis

Double-fluorescence quantitative video microscopy was used to measure the level of Pt–DNA adducts. At least 50–100 nuclei per slide were processed for FITC and Hoechst fluorescence. The Hoechst image served a twofold purpose. Firstly, it was used to separate the nuclei from the background, using an automatic local threshold selection method derived by Kittler et al (1985). Secondly, it was used to provide a measure of the local DNA surface density for each point within each nucleus. A new approach to multiple fluorescence image analysis, fluorescence linear fit microscopy (FLFM), was developed, which allowed the quantitation of Pt–DNA adducts. Per nucleus, a linear fit of the FITC fluorescence signal at each point as a function of the local DNA surface density was performed. The resulting slope yields the portion of FITC fluorescence attributable to the presence of DNA and hence to the presence of Pt–DNA adducts. The intercept

© Cancer Research Campaign 1997

**Table 1** Adduct recognition of the GPt antibody

| Adduct | Determined by | Sensitivity |
|---|---|---|
| Monofunctional adduct | Isolated monofunctional adduct | Not detectable |
| Intrastrand AG adduct | Isolated intrastrand AG adduct<br>Poly(dA–dG).(dC–dT), platination level 14% | Not detectable<br>IA50[a] = 0.5 ng Pt ml$^{-1}$ |
| Intrastrand GG adduct | Isolated intrastrand GG adduct<br>Poly(dG).poly(dC), platination level 25% | IA20[b] = 0.8 ng Pt ml$^{-1}$<br>IA50 = 0.5 ng Pt ml$^{-1}$ |
| Intrastrand adduct | Isolated interstrand adduct<br>Poly(dG–dC).poly(dG–dC), platination level 9% | IA20 = 3.75 ng Pt ml$^{-1}$<br>IA50 = > 500 ng Pt ml$^{-1}$ |

[a]IA50, amount of inhibitor with 50% inhibition in the competitive ELISA; [b]IA20, amount of inhibitor with 20% inhibition in the competitive ELISA.

**Figure 1** Immunostaining of $GLC_4$ showed as combined FITC and Hoechst images. Upper left, control cells (antibody background); upper right, cells after a 4-h incubation with 3.3 μM CDDP; lower left, cells after a 4-h incubation with 16.5 μM CDDP; lower right, cells after a 4-h incubation with 33 μM CDDP

**Figure 2** Immunostaining of $GLC_4$-CDDP showed as combined FITC and Hoechst images. Upper left, control cells (antibody background); upper right, cells after a 4-h incubation with 3.3 μM CDDP; lower left, cells after a 4-h incubation with 16.5 μM CDDP; lower right, cells after a 4-h incubation with 33 μM CDDP

yields the background fluorescence that is not attributable to DNA or Pt–DNA adducts. A goodness-fit measure (mean absolute deviates from the model must be less than 5) was used to detect and eliminate cells in which the linear fit was disturbed automatically. The median immunosignal showed a good correlation with the exposure time used (data not shown). Therefore, when necessary, the immunosignal was corrected for exposure time and/or nucleus size. Once corrected, the immunosignal should be a linear function of the amount of Pt–DNA adducts in each nucleus. Two different kinds of saturation effects may occur: firstly, camera saturation (overexposure), which can be avoided by appropriate adjustment of exposure time; secondly, intrinsic saturation of the fluorescence signal, can be the result of sterical hindrance, limiting the accessibility of the antibodies to their targets, and of fluorophore–fluorophore interactions at high dye concentrations. In case of in vitro experiments, this can be avoided by adjustment of the extracellular drug incubation concentrations. The use of a negative and a positive standard control guaranteed comparable quantitation of Pt–DNA adducts. Pretreatment samples served as corresponding background controls. The fluorescence video microscopy system was based on personal computers equipped with image processing boards (MVP-AT, Matrox, Dorval Quebec) and Fairchild CCD-5000/1 cameras (Loral-Fairchild, Sunnyvale, CA, USA) as described previously (Wilkinson et al, 1993; 1994).

### Applicability of GPT

The applicability of GPt was evaluated in human cell lines, normal cells (buccal cells and/or lymphocytes) and tumour cells (tumour cell suspensions, imprints of fresh tumour biopsies or paraffin-embedded tumour biopsies) after CDDP exposure in vitro or in vivo. $GLC_4$ and its 10-fold CDDP-resistant subline $GLC_4$-CDDP (characterized by an unchanged cellular Pt level, an increased glutathione level, a decreased DNA platination and an increased DNA repair capacity; Hospers et al, 1988, 1990; Meijer et al, 1990) were used to test the immunocytochemical application of GPt in cell lines. [CDDP concentrations inhibiting cell survival by 50% after a 4-h incubation with CDDP were 3.0 and 27.3 μM respectively (Meijer et al, 1990).] Buccal cells and/or lymphocytes (isolated from heparinized blood with a lymphoprep gradient; density 1.077 g ml$^{-1}$ 20°C; Nycomed, Pharma AS, Oslo, Norway) were sampled, subsequently washed

© Cancer Research Campaign 1997

**Figure 3** Computer-analysed levels of Pt–DNA adducts in $GLC_4$ (O–O) and $GLC_4$-CDDP (●–●) after immunostaining with GPt. FITC exposure time, 1 s; mean nuclei size, 945 and 910 pixels for $GLC_4$ and $GLC_4$-CDDP respectively with RPMI/3% FCS, followed by direct cytospin preparation in the case of in vivo CDDP-exposed samples or incubation with CDDP for the in vitro experiments, followed by cytospin preparation for immunostaining. Fresh tumour samples taken before chemotherapy were divided into parts and incubated with CDDP. Thereafter, imprints were prepared for immunostaining. For the measurement of Pt–DNA adduct levels after in vitro CDDP exposure under similar conditions, samples were incubated for 4 h with 3.3, 16.5 and 33 µM CDDP followed by washing, preparation, fixation and storage of the slides at –20°C until immunostaining. Feasibility of predictive testing was evaluated under the described in vitro conditions in human testicular and colon tumours, representing, respectively, CDDP-sensitive and -resistant tumour types in the clinic.

In vivo Pt–DNA adduct levels were determined in normal buccal cells and/or tumour cells of four patients before, during and after treatment with CDDP-containing chemotherapy. Chemotherapy included 80–100 mg $m^{-2}$ CDDP administered either over 5 days ($n$ = 2) or as a bolus infusion with an interval of 3 weeks ($n$ = 2). Pt-DNA adducts were also measured in sections of paraffin-embedded tumour biopsies of patients who received hyperthermic isolated limb perfusion with CDDP for extremity tumours (CDDP dose ranged from 20 to 30 mg $l^{-1}$ extremity volume) (Guchelaar et al, 1992). Parallel sections were routinely stained with haematoxylin–eosin to check the presence of tumour cells.

**Figure 4** (**A**) Immunostaining of buccal cells after in vitro CDDP exposure, shown as combined FITC and Hoechst images. Upper left, control buccal cells (antibody background); upper right, buccal cells after a 4-h incubation with 3.3 µM CDDP; lower left, buccal cells after a 4-h incubation with 16.5 µM CDDP; lower right, buccal cells after a 4-h incubation with 33 µM CDDP. (**B**) The corresponding computer-analysed level of Pt–DNA adducts. FITC exposure time, 0.1 s; mean nuclei size, 422 pixels

### RESULTS

### Antibody development

Serum was selected because of its high binding capacity for low-level platinated DNA and its lack of binding to control DNA. GPt was derived after purification of selected serum with affinity chromatography. The detection limit of GPt in the ELISA was approximately 25 fmol of Pt per assay well, and stable detection could be observed up to a drug–nucleotide ratio of 0.001. GPt was evaluated with various platinated adducts (isolated from $GLC_4$) and DNA polymers to detect both the intrastrand Pt–GG adducts and interstrand cross-links in a pharmacologically relevant DNA platination area at the adduct level (competitive ELISA, Table 1).

© Cancer Research Campaign 1997

A

B

**Figure 5** (**A**) Immunostaining of lymphocytes after in vitro CDDP exposure, shown as combined FITC and Hoechst images. Upper left, control lymphocytes (antibody background); upper right, lymphocytes after a 4-h incubation with 3.3 μM CDDP; lower left, lymphocytes after a 4-h incubation with 16.5 μM CDDP; lower right, lymphocytes after a 4-h incubation with 33 μM CDDP. (**B**) The corresponding computer-analysed level of Pt–DNA adducts. FITC exposure time, 5 s; mean nuclei size, 390 pixels

### Immunocyto- and histochemical application of GPt: in vitro DNA platination

Figures 1 and 2 show the combined Hoechst and FITC signals of a representative immunostaining experiment in $GLC_4$ and $GLC_4$-CDDP respectively. Nuclear FITC fluorescence was almost negative in CDDP-untreated, GPt-treated cells (background control) and increased dose-dependently in both cell lines. Immunostaining followed by computer image analysis showed dose-response curves (Figure 3) in both $GLC_4$ and $GLC_4$-CDDP for the CDDP concentrations used and the median immunosignal of the nucleus. There was a two- to five-fold difference in the level of Pt–DNA adducts between the cell lines. Also carboplatin-induced DNA platination could be detected (data not shown). Based on available immunocytochemistry and atomic absorption spectrometry (AAS) data for $GLC_4$, the lower detection limit of the immunocytochemical assay approaches 10 fmol of Pt per μg of DNA. Reproducibility of the assay, tested by measuring Pt–DNA adduct levels in four individually performed staining experiments of $GLC_4$, was calculated to be within 25% (coefficient of variance). The observed saturation of fluorescence at high extracellular CDDP incubation concentrations is clearly intrinsic to the specimen and is not caused by camera saturation.

**Figure 6** Computer-analysed levels of Pt–DNA adducts, corrected for exposure time and differences in cell size, after in vitro CDDP exposure of human testicular tumours (○–○) and colon tumours (●–●). Mean ± s.e.m.; *n* = 3; FITC exposure time, 0.1–0.5 s; mean nuclei size, 790 and 772 pixels for the testicular and colon tumours respectively

Figures 4 and 5 show photographs of representative immunostaining experiments and the corresponding computer analysis of the level of Pt–DNA adducts in buccal cells and lymphocytes after in vitro CDDP exposure. In contrast to the buccal cells, which showed a high level of Pt–DNA adducts, lymphocytes showed a low level of immunofluorescence (FITC exposure times were 0.1 s for the buccal cells and 5 s for the lymphocytes). The level of Pt–DNA adducts (corrected for exposure time and cell size) after in vitro CDDP exposure was fivefold higher in human testicular tumours than in colon tumour imprints (Figure 6). Large differences in Pt–DNA adduct levels (corrected for exposure time and nuclei size) after in vitro CDDP exposure were observed between cell lines, buccal cells, lymphocytes and different tumour types (Figures 3–6).

© Cancer Research Campaign 1997

**Figure 7** Computer-analysed levels of Pt–DNA adducts, corrected for nuclei size of samples of normal buccal cells (○–○) as well as imprints of a lymph node (●–●) of a seminoma patient. Samples were taken before and on days 1–4 of a 5-day CDDP regimen (20 mg m$^{-2}$ CDDP per day) and 4 days after the end of treatment. FITC exposure time, 1 s

### In vivo DNA platination

Figure 7 shows in vivo Pt–DNA adduct levels measured in normal buccal cells and in smears of a lymph node. Samples were collected from a patient (patient A) with seminoma before and on days 1–4 of a 5-day CDDP regimen (20 mg m$^{-2}$ CDDP per day) and 4 days after the end of treatment. The level of Pt–DNA adducts increased during the treatment in buccal and tumour cells in this patient, who was cured by this treatment. The Pt–DNA adduct level declined in the buccal cells but was still detectable 4 days after the last administration of CDDP. Pt–DNA adducts were also measured in buccal cells of three other patients (Table 2). A non-increasing low level of Pt–DNA adducts measured over 5 days was observed in samples of a non-responding patient (patient B) with an extragonadal germ cell tumour. Increasingly high levels of Pt–DNA adducts were observed over two and three cycles (with an interval of 3 weeks) of CDDP-containing chemotherapy in two responding patients with choriocarcinoma (patients C and D). Pt–DNA adducts were also measured in paraffin-embedded human tumour samples after hyperthermic isolated limb perfusion with CDDP. Pt–DNA adduct levels, expressed as median immunosignal after 1 s FITC exposure time, were 0.41 ($n$ = 1) immediately after, 1.31 ($n$ = 3) 3–6 weeks after and 0.76 ($n$ = 3) 3 months after perfusion. These levels were clearly detectable and were similar to the Pt–DNA adduct level of $GLC_4$ after a 4-h exposure to 3.3 μM CDDP. There was still a significant level of Pt–DNA adducts detectable 3 months after CDDP perfusion.

**Table 2** In vivo Pt–DNA adduct levels determined in normal buccal cells before, during and after chemotherapy. Chemotherapy included 80–100 mg m$^{-2}$ CDDP administered over 5 days (**A**) or as a bolus infusion with an interval of 3 weeks (**B**)

**A**

| Time in days | Patient A | Patient B |
|---|---|---|
| 1 | Negative (–, $n$ = 71)[a] | Not determined |
| 2 | 2.28 (16%, $n$ = 60) | 1.55 (25%, $n$ = 57) |
| 3 | 3.01 (11%, $n$ = 90) | 1.66 (13%, $n$ = 63) |
| 4 | 3.45 (15%, $n$ = 77) | 0.50 (36%, $n$ = 64) |
| 5 | Not determined | 1.71 (24%, $n$ = 65) |
| 8 | 0.18 (28%, $n$ = 51) | Not determined |

**B**

| Cycles of 3 weeks | Patient C | Patient D |
|---|---|---|
| 1 | 1.97 (25%, $n$ = 62) | 1.37 (16%, $n$ = 52) |
| 2 | 11.7 (16%, $n$ = 58) | 0.85 (11%, $n$ = 67) |
| 3 | Not determined | 4.85 (19%, $n$ = 57) |

[a]Median immunosignal of the nucleus (standard error in percentage, number of nuclei determined). FITC exposure time, 5 s.

### DISCUSSION

The existence of natural resistance or the development of acquired resistance to CDDP is the major cause of treatment failure with this drug in solid tumours. Studies in a variety of cell lines have revealed that several mechanisms can be involved in resistance to CDDP, including decreased drug accumulation, increased detoxification, decreased DNA platination and/or increased DNA repair (Bedford et al, 1988; Andrews and Howell, 1990; Fry et al, 1991; Sark et al, 1995). In almost all models, a combination of mechanisms is found, often resulting in a reduced DNA platination. Current information, however, suggests that not only the occurrence of adducts but also their quantity determine the fate of the cell. Another important factor may be the degree of tolerance to such damage or alternatively the occurrence of apoptotic cell death.

For a long time, evaluation of the relationship between in vivo DNA platination and response to chemotherapy has been limited by the requirement of large quantities of human material for reliable measurements of DNA platination by AAS. The development of antisera against platinated DNA has opened the way for the detection of low-level Pt–DNA adducts in human material (Poirier et al, 1982; Lippard et al, 1983; Fichtinger-Schepman et al, 1985; Plooy et al, 1985; Sundquist et al, 1987; Terheggen et al, 1987, 1991; Tilby et al, 1991; Chao et al, 1994). Until now, no serial measurements of Pt–DNA adducts have been performed in tumour cells of CDDP-treated patients.

In the present study, we describe the adaptation of a routine immunocyto- and histochemical staining protocol using GPt, the newly developed polyclonal antibody against platinated DNA, allowing sensitive detection of Pt–DNA adducts at a single-cell level in small samples of a broad range of material. Moreover, the morphological localization of Pt–DNA adducts with this technique allows analysis of tumour tissue and normal tissue. GPt, derived after immunization of rabbits with highly CDDP-platinated human DNA and subsequent purification of serum by affinity chromatography, was shown to detect the main Pt-containing intrastrand (Pt-GG) and interstrand adducts. An immunocyto- and histochemical technique was developed that allows the detection of low-level Pt–DNA adducts. Multiple fluorescence video image analysis, FLFM, was successfully used to quantitate the Pt–DNA adducts.

© Cancer Research Campaign 1997 

In the cell lines $GLC_4$ and $GLC_4$-CDDP, the slopes derived from FLFM correlated well with the more common and easier form of fluorescence quantitation, i.e. by measuring mean surface fluorescence per nucleus. Although simpler to implement, the latter method could not measure, and thereby correct for, background fluorescence in more complicated slides, such as paraffin sections, and moreover could not automatically eliminate objects (where the fluorescence model breaks down, by use of a goodness-fit measure (data not shown).

Differences in Pt–DNA adduct levels after in vitro CDDP exposure between CDDP-sensitive and- resistant cell lines could be clearly detected. DNA platination measurements with AAS showed a 1.6-fold difference in DNA platination between $GLC_4$ and the CDDP resistant $GLC_4$-CDDP at the highest CDDP incubation concentration used in the present study (Meijer et al, 1990). Based on the available immunocytochemistry and AAS results for $GLC_4$, the detection limit of our immunocytochemical assay approached 10 fmol of Pt per μg of DNA [approximately 0.2 amol of Pt per genome (nucleus)], which should allow the measurement of Pt–DNA adducts after exposure to CDDP in vivo (Terheggen et al, 1988). The at least 10-fold higher sensitivity compared with AAS and the possibility to measure Pt–DNA adducts in only a few cells (100–150 cells compared with $1–5 \times 10^7$ cells needed for reliable AAS measurements) are important advantages and requirements for the determination of Pt–DNA adducts in biological (patient) samples.

In vitro CDDP exposure revealed high platination levels in buccal cells compared with lymphocytes. Terheggen et al (1988) also reported a lower level of Pt–DNA adducts in human lymphocytes than in buccal cells after in vitro CDDP exposure. Apart from differences in cell viability, no explanation for this difference in the level of in vitro Pt–DNA adducts between human buccal cells and lymphocytes seems to be available. Pt–DNA adduct levels after in vitro exposure of leucocytes to CDDP were found to correlate with Pt–DNA adduct levels of leucocytes obtained from the same patient after CDDP treatment (Fichtinger–Schepman et al, 1990). In the present study, in vitro CDDP exposure of samples from different human tumour types showed fivefold higher Pt–DNA adduct levels in testicular tumours than in colon carcinomas. This suggests the possibility of predictive testing for this method. A confirmatory study is required in which a variety of tumours with known different degrees of sensitivity to CDDP should be included.

Pt–DNA adduct levels in patients has, until now, predominantly been studied by three groups, but only in leucocytes and never in tumour cells of patients before, during or after Pt-containing chemotherapy. The largest studies have been performed by Reed and Poirier in over 100 patients with ovarian and/or testicular cancer. In their studies, the quantity of measurable Pt–DNA adducts in leucocytes, determined with ELISA in most cases, increased as a function of cumulative dose of CDDP during repeated daily infusion of the drug and over a longer period with repeated cycles of administration. Pt–DNA adduct formation was consistently and directly related to therapy response (Poirier et al, 1985, 1987, 1992; Reed et al, 1986, 1987, 1988, 1990; Parker et al, 1991). More recently, this correlation has also been shown for patients with 24 different types of malignancies. This suggests that leucocytes may process DNA platination in a similar way to the tumour, regardless of the tissue of origin of the tumour (Dabholkar et al, 1992; Reed et al, 1993). Furthermore, similar levels of DNA platination have been reported in tumour and bone marrow tissue obtained at autopsy from Pt-treated patients (Reed et al, 1987; Poirier et al, 1992). The same studies, however, also showed differences in DNA platination between various organs and human tissues from the same patients. Fichtinger-Schepman et al (1985, 1989) used antibodies raised against synthetic haptens mimicking the Pt-containing digestion products of DNA to detect the various Pt–DNA adducts after chromatography of enzymatically digested DNA samples, also by use of the ELISA (Plooy et al, 1985). They also reported higher Pt–DNA adduct levels in the leucocytes of testicular cancer patients who showed a complete tumour response than in the leucocytes of those with a partial response or progressive disease. Substantial interindividual variation in Pt–DNA adduct levels after treatment was observed, correlating with Pt–DNA adduct levels obtained after in vitro CDDP exposure of leucocytes from the same patients sampled before treatment (Fichtinger-Schepman et al, 1990). Den Engelse et al used immunodensitometry to study Pt–DNA adduct levels in buccal cells after CDDP and/or carboplatin exposure (Terheggen et al, 1988; Gill et al, 1991; Blommaert et al, 1993). In addition, for buccal cells, large interindividual differences in Pt–DNA adduct levels were reported after carboplatin and/or CDDP treatment. Pt–DNA adduct levels were found to be higher in partial responders than in non-responders (Blommaert et al, 1993). Human buccal cells incubated in vitro with CDDP showed linear relationships between Pt–DNA adduct levels and either CDDP concentration or incubation time. No quantitative correlation, however, was found between in situ and in vitro Pt–DNA adduct levels (Terheggen et al, 1988).

Only Fichtinger-Schepman et al (1990) have reported a case of Pt–DNA adduct levels during treatment. They measured Pt–DNA adducts in DNA isolated from a testicular tumour 3 days after the first cycle of a 5-day CDDP regimen. The Pt–GG adduct level in tumour tissue was 10-fold higher than in leucocytes during CDDP treatment (Fichtinger-Schepman et al, 1990).

In the present study, only small series of samples were evaluated for Pt–DNA adducts after treatment with CDDP in vivo. Pt–DNA adducts could be clearly detected in buccal cells, tumour cells and paraffin-embedded tumour biopsies after CDDP-containing therapy and moreover, as far as was evaluable, was found to be correlated to response to chemotherapy. Additional information on this subject, focusing on the quantity of Pt–DNA adducts after in vivo exposure or the level of Pt–DNA adducts after in vitro CDDP exposure as a measure for therapy response in readily accessible tumour tissues, is however needed. The method described in this study allows analysis of low-level Pt–DNA adducts at a single-cell level in small samples after both in vitro and in vivo exposure to CDDP and, therefore, may allow the measurement of the amount of Pt–DNA adducts required to predict response to CDDP-based therapy.

### ACKNOWLEDGEMENTS

The authors like to thank Judy Bun and Petra Visser for technical assistance. This study was supported by grants GUKC 90-18 and 91-09 of the Dutch Cancer Foundation.

### ABBREVIATIONS

CDDP, cisplatin; Pt, platinum; FCS, fetal calf serum; mBSA, methylated bovine serum albumin; Hoechst, Hoechst 33258; FITC, fluorescein isothiocyanate; ELISA, enzyme-linked immunosorbent assay; PBS-1, phosphate-buffered saline 1 (0.14 M sodium chloride,

© Cancer Research Campaign 1997

2.7 mM potassium chloride, 6.4 mM disodium hydrogen phosphate, 1.5 mM potassium hydrogen phosphate, pH 7.4); PBS-2, phosphate-buffered saline 2 (0.15 M sodium chloride, 7.7 mM disodium hydrogen phosphate, 1.6 mM potassium hydrogen phosphate, pH 7.4); FLFM, fluorescence linear fit microscopy; AAS, atomic absorption spectrometry

### REFERENCES

Andrews PA and Howell SB (1990) Cellular pharmacology of cisplatin: perspectives on mechanisms of acquired resistance. *Cancer Cells* **2**: 35–43

Bedford P, Fichtinger-Schepman AMJ, Shellard SA, Walker C, Masters JRW and Hill BT (1988) Differential repair of platinum-DNA adducts in human bladder and testicular tumour continuous cell lines. *Cancer Res* **48**: 3019–3024

Blommaert FA, Michael C, Terheggen PMAB, Muggia FM, Kortes V, Schornagel JH, Hart AAM and Den Engelse L (1993) Drug-induced DNA modification in buccal cells of cancer patients receiving carboplatin and cisplatin combination chemotherapy, as determined by an immunocytochemical method: interindividual variations and correlation with disease response. *Cancer Res* **53**: 5669–5675

Chao CC-K, Shieh T-C and Huang H (1994) Use of a monoclonal antibody to detect DNA damage caused by the anticancer drug cis-diamminedichloroplatinum(II) in vivo and in vitro. *FEBS Lett* **354**: 103–109

Dabholkar M, Bradshaw L, Parker RJ, Gill I, Bostick-Bruton F, Muggia FM and Reed E (1992) Cisplatin-DNA damage and repair in peripheral blood leukocytes in vivo and in vitro. *Environmental Health Perspectives* **98**: 53–59

Fichtinger-Schepman AMJ, Baan RA, Luiten-Schuite A, Van Dijk M and Lohman PHM (1985) Immunochemical quantitation of adducts induced in DNA by cis-diamminedichloroplatinum(II) and analysis of adducts-related DNA-unwinding. *Chem Biol Interactions* **55**: 275–288

Fichtinger-Schepman AMJ, Van Oosterom AT, Lohman PHM and Berends F (1987) Cisplatin-induced DNA adducts in peripheral leucocytes from seven cancer patients; quantitative immunochemical detection of the adduct induction and removal after a single dose of cisplatin. *Cancer Res* **47**: 3000–3004

Fichtinger-Schepman AMJ, Baan RA and Berends F (1989) Influence of the degree of DNA modification on the immunochemical determination of cisplatin-DNA adduct levels. *Carcinogenesis* **10**: 2367–2369

Fichtinger-Schepman AMJ, Van der Velde-Visser SD, Van Dijk-Knijnenburg HCM, Van Oosterom AT, Baan RA and Berends F (1990) Kinetics of the formation and removal of cisplatin-DNA adducts in blood cells and tumour tissue of cancer patients receiving chemotherapy: comparison with in vitro adduct formation. *Cancer Res* **50**: 7887–7894

Fry AM, Chresta CM, Davies SM, Walker MC, Harris JAL, Hartley JA, Masters JR and Hickson ID (1991) Relationship between topoisomerase II level and chemosensitivity in human tumour cell lines. *Cancer Res* **51**: 6592–6595

Gill I, Muggia FM, Terheggen PMAB, Michael C, Parker RJ, Kortes V, Grunberg S, Christian MC, Reed E and Den Engelse L (1991) Dose-escalation study of carboplatin (day 1) and cisplatin (day 3): tolerance and relation to leukocyte and buccal cell platinum-DNA adducts. *Ann Oncol* **2**: 115–121

Guchelaar H-J, Hoekstra HJ, De Vries EGE, Uges DRA, Oosterhuis JW and Schraffordt Koops H (1992) Cisplatin and platinum pharmacokinetics during hyperthermic isolated limb perfusion for human tumours of the extremities. *Br J Cancer* **65**: 898–902

Hamburger AW and Salmon SE (1977) Primary bioassay of human tumour stem cells. *Science* **197**: 461–463

Hospers GAP, Mulder NH, De Jong B, De Leij L, Uges DRA, Fichtinger-Schepman AMJ, Scheper RJ and De Vries EGE (1988) Characterization of a human small cell lung carcinoma cell line with acquired cisplatin resistance in vitro. *Cancer Res* **48**: 6803–6807

Hospers GAP, Meijer C, De Leij L, Uges DRA, Mulder NH and De Vries EGE (1990) A study of human small cell lung carcinoma (hSCLC) cell lines with different sensitivities to detect relevant mechanisms of cisplatin (CDDP) resistance. *Int J Cancer* **46**: 138–144

Kittler J, Illingworth J and Föglein J (1985) Threshold selection based on a simple image statistic. *Comp Vision Graph Image Proc* **30**: 125–147

Lippard SJ, Ushay HM, Merkel CM and Poirier MC (1983) Use of antibodies to probe the stereochemistry of antitumour platinum drug binding to deoxyribonucleic acid. *Biochemistry* **22**: 5165–5168

Loehrer PJ and Einhorn LH (1984) Cisplatin. *Ann Intern Med* **100**: 704–713

Meijer C, Mulder NH, Hospers GAP, Uges DRA and De Vries EGE (1990) The role of glutathione in resistance to cisplatin in a human small cell lung cancer cell line. *Br J Cancer* **62**: 72–77

Mosman T (1983) Rapid colorimetric assay for cellular growth and survival: application to proliferation and cytotoxicity assays. *J Immunol Methods* **65**: 55–63

Parker RJ, Gill I, Tarone R, Vionnet JA, Grunberg S, Muggia FM and Reed E (1991) Platinum-DNA damage in leukocyte DNA of patients receiving carboplatin and cisplatin chemotherapy, measured by atomic absorption spectrometry. *Carcinogenesis* **12**: 1253–1258

Pieters R, Huismans DR, Loonen AH, Hählen K, Van der Does-van den Berg A, Van Wering ER and Veerman AJP (1991) Relation of cellular drug resistance to long-term clinical outcome in childhood acute leukaemia. *Lancet* **338**: 399–403

Plooy ACM, Fichtinger-Schepman AMJ, Schutte HH, Van Dijk M and Lohman PHM (1985) The quantitative detection of various Pt-DNA-adducts in Chinese hamster ovary cells treated with cisplatin: application of immunochemical techniques. *Carcinogenesis* **6**: 561–566

Poirier MC, Lippard SJ, Zwelling LA, Ushay HM, Kerrigan D, Thill CC, Santella RM, Grunberger D and Yuspa SH (1982) Antibodies elicited against cis-diamminedichloroplatinum(II)-modified DNA are specific for cis-diammine-dichloroplatinum(II)-DNA adducts formed in vivo and in vitro. *Proc Natl Acad Sci USA* **79**: 6443–6447

Poirier MC, Reed E, Zwelling LA, Ozols RF, Litterst CL and Yuspa SH (1985) Polyclonal antibodies to quantitate cis-diamminedichloro-platinum(II)-modified DNA adducts in cancer patients and animal models. *Environmental Health Perspectives* **62**: 89–94

Poirier MC, Reed E, Ozols RF, Fasy T and Yuspa SH (1987) DNA adducts of cisplatin in nucleated peripheral blood cells and tissues of cancer patients. *Prog Exp Tumour Res* **31**: 104–113

Poirier MC, Reed E, Litterst CL, Katz D and Gupta-Burt S (1992) Persistence of platinum-ammine-DNA adducts in gonads and kidneys of rats and multiple tissues from cancer patients. *Cancer Res* **52**: 149–153

Reed E, Yuspa SH, Zwelling LA, Ozols RF and Poirier MC (1986) Quantitation of cis–diamminedichloroplatinum II (cisplatin)-DNA-intrastrand adducts in testicular and ovarian cancer patients receiving cisplatin chemotherapy. *J Clin Invest* **77**: 545–550

Reed E, Ozols RF, Tarone R, Yuspa SH and Poirier MC (1987) Platinum-DNA adducts in leucocyte DNA correlate with disease response in ovarian cancer patients receiving platinum-based chemotherapy. *Proc Natl Acad Sci USA* **84**: 5024–5028

Reed E, Ozols RF, Tarone R, Yuspa SH and Poirier MC (1988) The measurement of cisplatin–DNA adduct levels in testicular cancer patients. *Carcinogenesis* **9**: 1909–1911

Reed E, Ostchega Y, Steinberg SM, Yuspa SH, Young RC, Ozols RF and Poirier MC (1990) Evaluation of platinum-DNA adduct levels relative to known prognostic variables in a cohort of ovarian cancer patients. *Cancer Res* **50**: 2256–2260

Reed E, Parker RJ, Gill I, Bicher A, Dabholkar M, Vionnet JA, Bostick-Bruton F, Tarone R and Muggia FM (1993) Platinum-DNA adduct in leukocyte DNA of a cohort of 49 patients with 24 different types of malignancies. *Cancer Res* **53**: 3694–3699

Sark MWJ, Timmer-Bosscha H, Meijer C, Uges DRA, Sluiter WJ, Peters WHM, Mulder NH and De Vries EGE (1995) Cellular basis for differential sensitivity to cisplatin in human germ cell tumour and colon carcinoma cell lines. *Br J Cancer* **71**: 684–690

Sundquist WI, Lippard SJ and Stollar BD (1987) Monoclonal antibodies to DNA modified with cis- or trans-diamminedichloroplatinum(II). *Proc Natl Acad Sci USA* **84**: 8225–8229

Terheggen PMAB, Floot BGL, Scherer E, Begg AC, Fichtinger-Schepman AMJ and Den Engelse L (1987) Immunocytochemical detection of interaction products of cis-diamminedichloroplatinum(II) and cis-diammine(1,1-cyclobutanedicarboxylato)platinum(II) with DNA in rodent tissue sections. *Cancer Res* **47**: 6719–6725

Terheggen PMAB, Dijkman R, Begg AC, Dubbelman R, Floot BGJ, Hart AAM and Den Engelse L (1988) Monitoring of interaction products of cis-diamminedichloroplatinum(II) and cis-diammine (1,1-cyclo-butanedicarboxylato)-platinum(II) with DNA in cells from platinum-treated cancer patients. *Cancer Res* **48**: 5597–5603

Terheggen PMAB, Floot BGJ, Lempers ELM, Van Tellingen O, Begg AC and Den Engelse L (1991) Antibodies against cisplatin-modified DNA and cisplatin-modified (di)nucleotides. Cancer Chemother Pharmacol **28**: 185–191

Tilby MJ, Johnson C, Knox RJ, Cordell J, Roberts JJ and Dean CJ (1991) Sensitive detection of DNA modifications induced by cisplatin and carboplatin in vitro and in vivo using a monoclonal antibody. *Cancer Res* **51**: 123–129

Weisenthal LM, Marsden JA, Dill PL and Macaluso CK (1983) A novel dye exclusion method for testing in vitro chemosensitivity of human tumours. *Cancer Res* **43**: 749–757

© Cancer Research Campaign 1997

Wilkinson MHF, Jansen GJ and Van der Waaij D (1993) Very low level fluorescence detection and imaging using a long exposure charge coupled device system. In *Biotechnology applications of microinjection, microscopic imaging and fluorescence*. Bach PH, Reynolds CH, Clark JM, Poole PL and Mottley J. (eds.), pp. 221–230. Plenum Press: New York

Wilkinson MHF, Jansen GJ and Van der Waaij D (1994) Computer processing of microscopic images of bacteria: morphometry and fluorimetry. *Trends Microbiol* 2: 485–489

© Cancer Research Campaign 1997